# Associations between serum amino acids and genetic liability to depression in the LURIC cohort

**DOI:** 10.1186/s12888-026-08400-7

**Published:** 2026-07-17

**Authors:** Robert M. Krämer, Graciela E. Delgado, Marcus E. Kleber, Daniel Duerschmied, Winfried März, Angela Patricia Moissl-Blanke

**Affiliations:** 1 Department for Children and Adolescent Psychiatry, Central Institute for Mental Health Mannheim, Medical Faculty Mannheim, University of Heidelberg, Mannheim, Germany; 2 Department of Medicine I (Cardiology, Hemostaseology, Medical Intensive Care), Medical Faculty Mannheim, University of Heidelberg, Mannheim, Germany; 3 Synlab Medical Center of Human Genetics, SYNLAB Holding Deutschland GmbH, Mannheim, Germany; 4 Synlab Academy, SYNLAB Holding Deutschland GmbH, Mannheim, Germany; 5 Department of Medicine III (Cardiology, Pneumology, Angiology), University of Heidelberg, Heidelberg, Germany

**Keywords:** Amino acid, Depression, Genetic risk score

## Abstract

**Aims:**

Blood concentrations of amino acids have been associated with depression, although findings have been inconsistent. We investigated associations between serum amino acid concentrations and genetic liability to depression, assessed by two genetic depression risk scores, in the Ludwigshafen Risk and Cardiovascular Health (LURIC) study.

**Methods:**

The LURIC study enrolled 3,316 participants referred for coronary angiography; serum amino acid measurements were available for 2,143 individuals and were quantified by ion-exchange chromatography. Two weighted genetic depression risk scores, based on 101 and 220 genome-wide significant variants, were calculated. Associations between genetic depression risk scores and amino acid concentrations were assessed using multivariable linear regression models adjusted for age, sex, body mass index, renal function (eGFR), diabetes, hypertension, and coronary artery disease. Clinical depression data were not available; thus, analyses reflect genetic liability rather than manifest depression.

**Results:**

In multivariable-adjusted analyses, higher genetic depression risk (per SD increase) was associated with lower concentrations of α-aminoadipic acid (GDRS₁₀₁: β = −0.097, 95% CI − 0.161 to − 0.033, *p* = 0.003; GDRS₂₂₀: β = −0.076, 95% CI − 0.141 to − 0.011, *p* = 0.021), which remained significant after false discovery rate (FDR) correction. α-aminobutyric acid showed inverse associations that did not remain significant after FDR correction. No statistically significant associations for other amino acids remained after multivariable adjustment and correction for multiple testing. Restricted cubic spline analyses suggested predominantly linear relationships, with evidence of non-linearity observed for selected amino acids, particularly homoarginine with respect to GDRS₂₂₀ (p for non-linearity = 0.005).

**Conclusion:**

Genetic liability to depression was associated with differences in serum amino acid concentrations, particularly lower α-aminoadipic acid concentrations in this cardiovascular cohort, whereas associations with other amino acids were not robust after correction for multiple testing. These findings are associative and hypothesis-generating and should be confirmed in cohorts with detailed depression phenotyping and longitudinal follow-up.

**Supplementary Information:**

The online version contains supplementary material available at 10.1186/s12888-026-08400-7.

## Introduction

Major depression is a common psychiatric disorder affecting approximately 7.5% of men and 13.6% of women worldwide, with a cumulative lifetime risk at age 75 of 20.1% in men and 34.0% in women [1]. The incidence peaks between 15 and 20 years of age in both sexes and declines thereafter.⁠ [1] According to the Global Burden of Disease Study 2017, depression ranks among the leading causes of years lived with disability globally, with a substantial increase in burden over recent decades [2]. 

Reliable biomarkers are urgently needed in mood disorders to improve diagnostic classification, monitor treatment response, and support the development of novel therapeutic strategies [3]. Among potential candidates, circulating amino acids such as glutamate, γ-aminobutyric acid (GABA), and tryptophan have been proposed as biologically relevant markers, given their roles in neurotransmission and metabolic regulation [3]. 

Numerous studies have examined amino acid profiles in major depression; however, findings have been inconsistent. While several cohort studies reported increased plasma concentrations of glutamate, aspartate, glycine, and taurine, others observed decreased concentrations of tryptophan, methionine, glutamine, and kynurenine.⁠ [4–7] Additional studies have identified reduced tryptophan, lysine, and GABA concentrations [8], whereas others reported increased concentrations of alanine, methionine, and GABA, or no differences in selected amino acids [7, 9–11]. Meta-analyses support reduced plasma tryptophan concentrations and elevated glutamate concentrations as relatively consistent findings, whereas arginine, ornithine, and citrulline appear less consistently associated.⁠ [12–15] Taken together, these findings suggest that alterations in amino acid metabolism—particularly within glutamatergic signalling and the tryptophan–kynurenine pathway—are associated with depression, although results remain heterogeneous [16–18]. 

However, the magnitude and direction of these associations vary substantially across studies, likely reflecting differences in study design, population characteristics, and analytical approaches. Beyond phenotypic associations, genetic approaches may provide complementary insights into the biological underpinnings of depression by capturing inherited susceptibility independent of current disease status. We have previously demonstrated in the Ludwigshafen Risk and Cardiovascular Health (LURIC) study that a genetic depression risk score (GDRS) is independently associated with all-cause and cardiovascular mortality [19, 20]. 

Given that circulating amino acid concentrations have also been linked to cardiovascular and metabolic diseases [21–23], we investigated the associations between genetic liability to depression and serum amino acid concentrations in participants of the LURIC cohort. In the absence of clinical depression phenotyping, our analyses focus on metabolic correlates of genetic liability to depression rather than manifest disease. We hypothesised that specific circulating amino acids are associated with genetic depression risk in this well-characterised cardiovascular population.

## Materials and methods

### Subjects

The Ludwigshafen Risk and Cardiovascular Health (LURIC) study is a monocentric, hospital-based cohort that enrolled 3,316 patients of German ancestry from the region surrounding Ludwigshafen, Mannheim, and Heidelberg at the Ludwigshafen Heart Center in South-West Germany. All participants were referred for elective coronary angiography to evaluate established or suspected coronary artery disease (CAD). Patients with acute illnesses other than acute coronary syndromes, chronic non-cardiac diseases, or a history of malignancy within the previous five years were excluded [24]. 

The study was approved by the ethics committee of the Landesärztekammer Rheinland-Pfalz (LURIC, #837.255.97 [1394]) and conducted in accordance with the Declaration of Helsinki. All participants provided written informed consent.

### Laboratory methods and definition of clinical variables

**Fig. 1:**
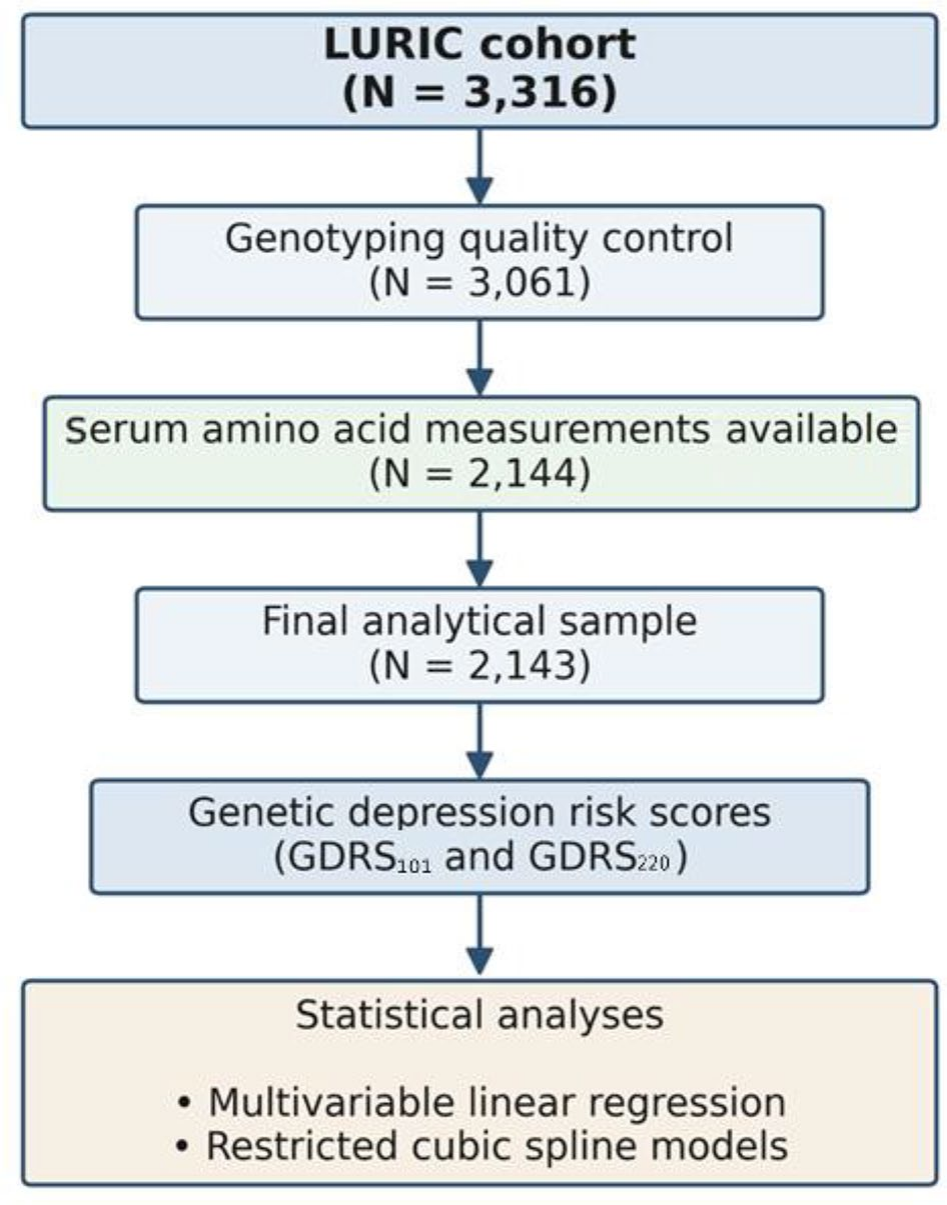
Flow diagram of the LURIC cohort illustrating participant inclusion and data availability for genetic depression risk scores (GDRS_101_ and GDRS_220_) and serum amino acid measurements

Fasting blood samples were obtained by venipuncture at study entry. Details of analytical procedures in the LURIC study have been described previously [24]. Serum amino acid concentrations (phosphoserine, taurine, aspartic acid, threonine, serine, asparagine, glutamic acid, glutamine, α-aminoadipic acid, proline, glycine, alanine, citrulline, α-aminobutyric acid, valine, cystine, methionine, isoleucine, leucine, norleucine, tyrosine, phenylalanine, histidine, tryptophan, ornithine, lysine, and arginine) were quantified after separation by ion-exchange chromatography with post-column ninhydrin derivatisation, as described previously [25, 26]. Homoarginine, asymmetric dimethylarginine (ADMA), and symmetric dimethylarginine (SDMA) were determined using reverse-phase high-performance liquid chromatography [25]. Samples were analysed after one year of storage at − 80 °C without prior thawing. Genetic data were available for 3,061 participants. Among these, amino acid measurements were available in 2,144 individuals; one participant was excluded due to incomplete data, resulting in 2,143 individuals included in the present analyses [24, 26]. A flow diagram of the study population is shown in Fig. 1.

Coronary artery disease (CAD) was defined according to the American Heart Association classification as the presence of > 20% lumen stenosis in at least one of 15 coronary segments [27]. Diabetes mellitus was defined according to the 2010 American Diabetes Association criteria [28]. Hypertension was defined as systolic blood pressure ≥ 140 mmHg, diastolic blood pressure ≥ 90 mmHg, or a history of hypertension [29]. Estimated glomerular filtration rate (eGFR) was calculated using the 2012 CKD-EPI creatinine–cystatin C equation [30]. 

### Genotyping, imputation and calculation of the genetic risk score of depression

Genotyping was performed using the Affymetrix Human SNP Array 6.0. Standard quality control procedures were applied, excluding samples with a call rate < 95% or sex discordance between genotypic and clinical data. Variants with a call rate < 98%, deviation from Hardy–Weinberg equilibrium (*p* < 5 × 10⁻⁴), or a minor allele frequency < 0.01 were excluded. Genotype imputation was performed using the 1000 Genomes Project Phase I v3 reference panel with IMPUTE2, as described previously [20]. 

Genetic depression risk scores were constructed based on previously published genome-wide association studies [19, 31]. Specifically, 101 of 102 genome-wide significant single-nucleotide polymorphisms (SNPs) from Howard et al. [19] and 220 of 223 SNPs from Levey et al. [31] were available and included. Weighted scores (GDRS₁₀₁ and GDRS₂₂₀) were calculated using PLINK v2.0 by summing the number of risk alleles at each locus, weighted by the corresponding effect sizes reported in the respective GWAS. Higher scores indicate greater genetic liability to depression.

### Statistical analyses

Continuous variables are presented as mean ± standard deviation (SD) for normally distributed data or as median (interquartile range) for skewed distributions. Categorical variables are reported as counts and percentages.

Genetic depression risk scores (GDRS₁₀₁ and GDRS₂₂₀) were analysed as continuous variables and standardised prior to analysis. All primary analyses and graphical representations were based on continuous, standardised GDRS to preserve statistical power and avoid information loss. The correlation between both scores was assessed using Spearman’s rank correlation and visualised in Supplementary Figure S1.

Associations between genetic depression risk scores and serum amino acid concentrations were assessed using multivariable linear regression models. Amino acid concentrations were log-transformed where appropriate to improve normality.

Primary models were adjusted for age, sex, body mass index (BMI), renal function (estimated glomerular filtration rate [eGFR]), diabetes mellitus, hypertension, and coronary artery disease. Additional adjustments for smoking status and medication were performed where data were available and did not materially alter the results. Effect estimates are reported as regression coefficients (β) with corresponding 95% confidence intervals (CI) per one standard deviation (SD) increase in the genetic depression risk score. To account for multiple testing, false discovery rate (FDR) correction was applied using the Benjamini–Hochberg procedure, and results were interpreted with emphasis on FDR-adjusted p-values. Non-linear associations between genetic depression risk scores and selected amino acids based on primary analyses were explored using restricted cubic spline regression models with knots placed at the 10th, 50th, and 90th percentiles. To account for overlap between GDRS₁₀₁ and GDRS₂₂₀, an exploratory principal component analysis (PCA) was performed as a sensitivity analysis. The first principal component explained 79% of the variance and was standardised to generate a combined genetic depression risk score (GDRS_combined), which was used in supplementary and sensitivity analyses.

All statistical tests were two-sided and performed using SPSS version 27 (IBM Corp., Armonk, NY, USA) and R version 4.3.1 (R Foundation for Statistical Computing, Vienna, Austria).

## Results

### Baseline characteristics of the LURIC cohort are summarised in Tables 1 and 2

**Table 1:** Baseline characteristics according to median split of the genetic depression risk score GDRS₁₀₁ (exploratory analysis)

Variable	Overall	Low GDRS	High GDRS	P*
	*N* = 2143	*N* = 1050	*N* = 1093	-
Age (years)	62.6 ± 10.8	62.9 ± 10.9	62.3 ± 10.7	0.535
Male sex (%)	70.2	70.1	70.3	0.822
BMI (kg/m^2^)	27.2 ± 3.9	27.21 ± 3.97	27.26 ± 3.88	0.978
LDL-Cholesterol (mg/dL)	115.4 ± 34.5	115.92 ± 35.01	114.94 ± 33.93	0.245
HDL-Cholesterol (mg/dL)	38.4 ± 10.7	38.45 ± 10.30	38.34 ± 11.15	0.628
Triglycerides (mg/dL)	146 (107.8–199)	146 (110–200)	147 (108–201)	0.542
HbA1c (%)	6.2 ± 1.2	6.3 ± 1.2	6.3 ± 1.3	0.542
Systolic Blood pressure (mmHg)	141.6 ± 23.7	141.29 ± 23.96	141.81 ± 23.47	0.788
Diastolic Blood pressure (mmHg)	80.6 ± 11.4	81.0 ± 11.2	80.70 ± 11.7	0.947
eGFR (mL/min/1.73m^2^)	82.8 ± 20.4	81.0 ± 20.4	82.0 ± 20.4	0.501
hsCRP (mg/L)	3.5 (1.3–8.7)	3.65 (1.34–9.02)	3.36 (1.31–8.48)	0.641
NT-proBNP (pg/mL)	294 (108–868.5)	302 (109–875)	292 (106–888)	0.732
Alcohol consumption (g eth/d)	14.8 ± 23.7	15.06 ± 23.6	14.6 ± 23.7	0.355
Coronary artery disease (%)	79.2	78.6	79.6	0.632
Hypertension (%)	72.9	71.6	74.4	0.184
Diabetes mellitus type 2 (%)	38.1	40.5	40.3	0.924
Smoking never/ex/active (%)	42.2/22.1/35.7	22/41/37.1	24/41/34.6	0.171

Values are presented as mean ± standard deviation (SD) for normally distributed variables, median (interquartile range) for skewed variables, or percentages for categorical variables. *P-values are derived from unpaired t-tests for continuous variables and χ² tests for categorical variables. A two-sided P-value < 0.05 was considered statistically significant. Median split analyses are provided for descriptive purposes only and were not used for primary inference. All main analyses were conducted using continuous, standardised GDRS variables

**Table 2:** Baseline characteristics according to median split of the genetic depression risk score GDRS₂₂₀ (exploratory analysis)

Variable	Overall	Low GDRS	High GDRS	P*
	*N* = 2143	*N* = 1065	*N* = 1078	-
Age (years)	62.6 ± 10.8	63.2 ± 10.7	61.9 ± 10.9	0.038
Male sex (%)	70.2	70.1	70.3	0.392
BMI (kg/m^2^)	27.2 ± 3.9	27.23 ± 3.88	27.24 ± 3.96	0.396
LDL-Cholesterol (mg/dL)	115.4 ± 34.5	115.8 ± 34.48	115.05 ± 34.45	0.389
HDL-Cholesterol (mg/dL)	38.4 ± 10.7	38.53 ± 10.84	38.26 ± 10.65	0.907
Triglycerides (mg/dL)	146 (107.8–199)	145 (108.5–195.5)	147.0 (106–202)	0.213
HbA1c (%)	6.2 ± 1.2	6.22 ± 1.14	6.21 ± 1.30	0.713
Systolic Blood pressure (mmHg)	141.6 ± 23.7	141.29 ± 23.61	141.81 ± 23.81	0.677
Diastolic Blood pressure (mmHg)	80.6 ± 11.4	80.21 ± 11.18	80.93 ± 11.66	0.281
eGFR (mL/min/1.73m^2^)	82.8 ± 20.4	81.01 ± 20.89	84.65 ± 19.73	0.001
hsCRP (mg/L)	3.5 (1.3–8.7)	3.55 (1.33–8.92)	3.42 (1.25–8.52)	0.657
NT-proBNP (pg/mL)	294 (108–868.5)	321.5 (117–899.3)	265 (100.5–822)	0.159
Alcohol consumption (g eth/d)	14.8 ± 23.7	14.52 ± 23.86	15.17 ± 23.46	0.427
Coronary artery disease (%)	79.2	78.9	79.6	0.172
Hypertension (%)	72.9	71.5	74.1	0.316
Diabetes mellitus type 2 (%)	38.1	39.2	37.1	0.292
Smoking never/ex/active (%)	42.2/22.1/35.7	37.1/42.6/20.3	34.4/41.7/23.9	0.078

Values are presented as mean ± standard deviation (SD) for normally distributed variables, median (interquartile range) for skewed variables, or percentages for categorical variables. *P-values are derived from unpaired t-tests for continuous variables and χ² tests for categorical variables. A two-sided P-value < 0.05 was considered statistically significant. Median split analyses are provided for descriptive purposes only and were not used for primary inference. All main analyses were conducted using continuous, standardised GDRS variables

The study population comprised 2,143 participants with available amino acid measurements. Overall, approximately 70% of participants were male, 79% had angiographically confirmed coronary artery disease, 73% had hypertension, and 40% had diabetes mellitus. The mean body mass index (BMI) was 27.2 kg/m². The two genetic depression risk scores (GDRS₁₀₁ and GDRS₂₂₀) were moderately correlated (Spearman’s *r* = 0.57, *p* < 0.001; Supplementary Figure S1), indicating overlapping but non-identical genetic architecture.

### Associations between genetic depression risk scores and amino acids

Associations between genetic depression risk scores and serum amino acid concentrations were assessed using multivariable linear regression models. Full regression results are presented in Supplementary Table S3. Unadjusted correlation analyses are provided in Supplementary Tables S1–S2 for descriptive purposes only and were not used for primary inference. Effect estimates are reported per one standard deviation (SD) increase in the respective genetic depression risk score.

In fully adjusted analyses, higher genetic depression risk was associated with lower concentrations of α-aminoadipic acid (GDRS₁₀₁: β = −0.097, 95% CI − 0.161 to − 0.033, *p* = 0.003; GDRS₂₂₀: β = −0.076, 95% CI − 0.141 to − 0.011, *p* = 0.021), with both associations remaining statistically significant after false discovery rate (FDR) correction.

Descriptive group-based comparisons showed broadly similar directional patterns for selected amino acids in Tables 3 and 4; however, these analyses were not used for primary inference due to potential information loss and susceptibility to confounding.

**Table 3:** Serum amino acid concentrations according to median split of GDRS₁₀₁ (exploratory analysis)

GDRS_101_	Low GDRS	High GDRS	P*	FDR
Variables	*N* = 1050	*N* = 1093	-	-
Aspartic acid, µmol/L	2.04 ± 3.3	1.90 ± 3.21	0.343	0.706
Threonine, µmol/L	121.0 ± 33.1	120.5 ± 32.5	0.812	0.922
Serine, µmol/L	96.2 ± 22.0	95.7 ± 22.5	0.615	0.896
Glutamic acid, µmol/L	80.9 ± 53.5	80.4 ± 51.2	0.818	0.922
Glycine, µmol/L	199.3 ± 56.5	198.2 ± 55.7	0.664	0.896
Valine, µmol/L	257.9 ± 53.9	255.5 ± 56.0	0.302	0.664
α-aminobutyric acid, µmol/L	23.1 ± 9.8	22.3 ± 9.4	0.041	0.664
Cystine, µmol/L	35.6 ± 29.3	34.1 ± 28.1	0.240	0.664
Methionine, µmol/L	25.3 ± 5.7	25.3 ± 5.9	0.851	0.922
Leucine, µmol/L	140.3 ± 31.7	138.5 ± 32.2	0.185	0.664
Isoleucine, µmol/L	71.2 ± 16.7	70.8 ± 17.2	0.604	0.896
Phenylalanine, µmol/L	58.7 ± 11.5	58.6 ± 12.7	0.894	0.922
3-Methylhistidine, µmol/L	2.56 ± 5.17	2.5 ± 5.0	0.726	0.921
1-Methylhistidine, µmol/L	5.22 ± 10.4	4.6 ± 9.1	0.119	0.664
Lysine, µmol/L	204.0 ± 42.1	203.2 ± 42.1	0.670	0.896
Asparagine, µmol/L	35.0 ± 11.6	35.23 ± 11.0	0.636	0.896
Glutamine, µmol/L	595.7 ± 119	601.6 ± 112.8	0.239	0.664
α-aminoadipic acid, µmol/L	0.32 ± 1.90	0.095 ± 0.983	0.001	0.018
Proline, µmol/L	192.3 ± 56.4	193.3 ± 57.3	0.670	0.896
Alanine, µmol/L	353.3 ± 88.4	353.9 ± 92.3	0.887	0.922
Citrulline, µmol/L	32.9 ± 11.8	33.5 ± 11.9	0.270	0.664
Norleucine, µmol/L	148.7 ± 7.3	149.2 ± 7.6	0.135	0.664
Tyrosine, µmol/L	65.3 ± 15.7	66.2 ± 17.0	0.174	0.664
Histidine, µmol/L	67.7 ± 24.8	66.8 ± 12.5	0.266	0.664
Tryptophan, µmol/L	40.6 ± 9.9	41.3 ± 14.3	0.240	0.664
Ornithine, µmol/L	58.2 ± 16.5	58.6 ± 16.7	0.569	0.896
Arginine, µmol/L	80.7 ± 20.0	82.0 ± 20.4	0.139	0.664
Kynurenin, µmol/L	2.8 ± 0.92	2.79 ± 0.897	0.787	0.922
ADMA, µmol/L	0.827 ± 0.154	0.825 ± 0.146	0.678	0.896
SDMA, µmol/L	0.588 ± 0.263	0.589 ± 0.232	0.944	0.944
Homoarginine, µmol/L	2.53 ± 1.07	2.59 ± 1.03	0.125	0.664
Taurine, µmol/L	64.5 ± 18.2	63.7 ± 19.3	0.300	0.664

Values are presented as mean ± standard deviation (SD). P-values are derived from unpaired t-tests. False discovery rate (FDR) correction was applied using the Benjamini–Hochberg procedure to account for multiple testing. Primary analyses were performed using multivariable regression models with continuous GDRS variables; group-based comparisons are provided for descriptive purposes only

Abbreviations: ADMA, asymmetric dimethylarginine; SDMA, symmetric dimethylarginine; GDRS, genetic depression risk score; FDR, false discovery rate

**Table 4:** Serum amino acid concentrations according to median split of GDRS₂₂₀ (exploratory analysis)

GDRS_220_	Low GDRS	High GDRS	P*	FDR
Variables	*N* = 1065	*N* = 1078	-	-
Aspartic acid, µmol/L	1.95 ± 3.23	1.98 ± 3.29	0.848	0.968
Homocysteine, µmol/L	13.6 ± 6.31	13.3 ± 5.7	0.091	0.459
Threonine, µmol/L	120.3 ± 33.9	121.2 ± 31.7	0.524	0.865
Serine, µmol/L	95.6 ± 22.0	96.3 ± 22.5	0.498	0.865
Methionine, µmol/L	25.2 ± 5.68	25.4 ± 5.9	0.271	0.788
Isoleucine, µmol/L	70.9 ± 16.9	71.04 ± 17.06	0.828	0.968
1-Methylhistidine, µmol/L	5.4 ± 10.7	4.4 ± 8.8	0.018	0.459
Asparagine, µmol/L	35.1 ± 11.3	35.1 ± 11.2	0.947	0.977
Glutamine, µmol/L	598.5 ± 117.1	599.0 ± 114.6	0.908	0.968
Proline, µmol/L	192.7 ± 56.4	192.95 ± 57.3	0.908	0.968
Alanine, µmol/L	350.6 ± 88.1	356.5 ± 92.6	0.127	0.524
Norleucine, µmol/L	149.04 ± 7.21	148.8 ± 7.7	0.502	0.865
Tyrosine, µmol/L	65.6 ± 16.2	65.9 ± 16.5	0.587	0.922
Tryptophan, µmol/L	40.7 ± 9.9	41.16 ± 14.35	0.387	0.798
Arginine, µmol/L	80.6 ± 20.2	82.09 ± 20.2	0.082	0.459
Glutamic acid, µmol/L	80.6 ± 52.6	80.6 ± 52.2	0.997	0.997
Glycine, µmol/L	199.3 ± 55.9	198.2 ± 56.3	0.659	0.937
Valine, µmol/L	257.2 ± 53.5	256.2 ± 56.4	0.670	0.937
α-aminobutyric acid, µmol/L	22.9 ± 9.8	22.5 ± 9.5	0.286	0.788
Leucine, µmol/L	139.4 ± 31.6	139.3 ± 32.3	0.910	0.968
Cystine, µmol/L	35.4 ± 29.2	34.3 ± 28.2	0.374	0.798
Phenylalanine, µmol/L	58.9 ± 12.1	58.4 ± 12.1	0.326	0.798
3-Methylhistidine, µmol/L	2.7 ± 5.4	2.7 ± 5.4	0.097	0.459
Lysine, µmol/L	203.9 ± 41.6	203.34 ± 42.61	0.768	0.968
α-aminoadipic acid, µmol/L	0.3 ± 1.7	0.15 ± 1.27	0.070	0.459
Citrulline, µmol/L	33.5 ± 12.5	32.9 ± 11.2	0.234	0.772
Histidine, µmol/L	67.3 ± 24.6	67.2 ± 12.6	0.889	0.968
Ornithine, µmol/L	58.6 ± 16.6	58.28 ± 16.6	0.682	0.937
Kynurenin, µmol/L	2.82 ± 0.97	2.77 ± 0.84	0.370	0.798
ADMA, µmol/L	0.83 ± 0.16	0.82 ± 0.15	0.170	0.623
SDMA, µmol/L	0.6 ± 0.28	0.58 ± 0.21	0.038	0.459
Homoarginine, µmol/L	2.53 ± 1.07	2.6 ± 1.03	0.059	0.459
Taurine, µmol/L	64.4 ± 18.6	63.8 ± 19.0	0.429	0.833

Values are presented as mean ± standard deviation (SD). P-values are derived from unpaired t-tests. False discovery rate (FDR) correction was applied using the Benjamini–Hochberg procedure to account for multiple testing. Primary analyses were performed using multivariable regression models with continuous GDRS variables; group-based comparisons are provided for descriptive purposes only

Abbreviations: ADMA, asymmetric dimethylarginine; SDMA, symmetric dimethylarginine; GDRS, genetic depression risk score; FDR, false discovery rate

α-aminobutyric acid showed inverse associations that did not remain statistically significant after FDR correction (GDRS₁₀₁: β = −0.404, 95% CI − 0.803 to − 0.005, *p* = 0.047; GDRS₂₂₀: β = −0.409, 95% CI − 0.813 to − 0.006, *p* = 0.047). No significant associations were observed for 1-methylhistidine (GDRS₁₀₁: β = −0.093, *p* = 0.637; GDRS₂₂₀: β = −0.166, *p* = 0.405).

Associations involving SDMA (GDRS₁₀₁: β = 0.002, *p* = 0.454; GDRS₂₂₀: β = 0.001, *p* = 0.717) and homoarginine (GDRS₁₀₁: β = 0.025, *p* = 0.154; GDRS₂₂₀: β = 0.006, *p* = 0.758) were not statistically significant in multivariable-adjusted models. Given the known dependence of these metabolites on renal function, these findings should be interpreted with caution despite adjustment for eGFR (CKD-EPI).

No further amino acids remained statistically significant after FDR correction.

### Non-linear associations

**Fig. 2:**
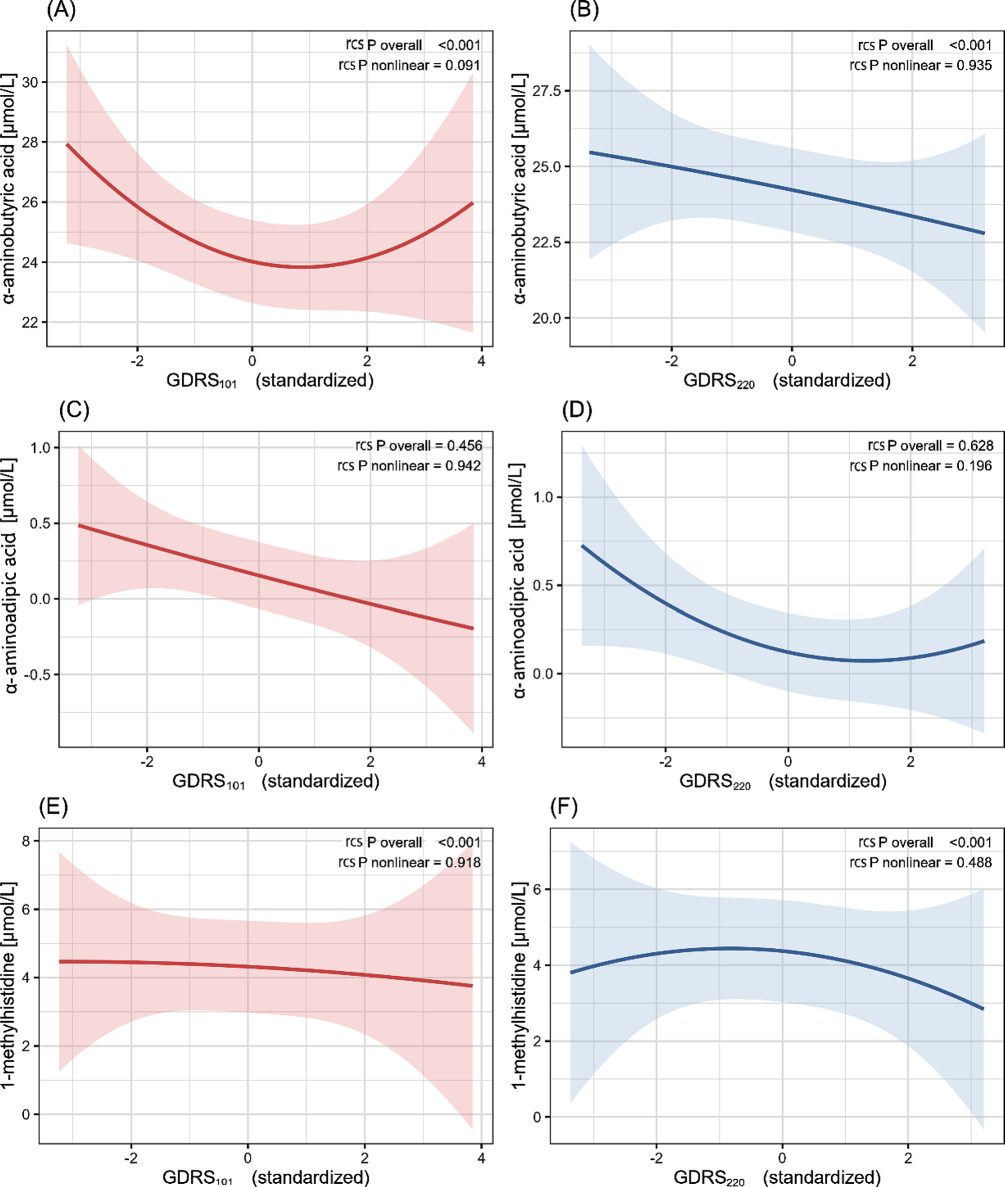
Associations between circulating amino acid concentrations and genetic depression risk scores assessed using restricted cubic spline (RCS) regression models. Panels (**A**), (**C**), and (**E**) depict associations with GDRS_101_, whereas panels (**B**), (**D**), and (**F**) depict associations with GDRS_220_. Solid lines represent model-based predicted values from multivariable-adjusted RCS models, and shaded areas indicate 95% confidence intervals. Models were adjusted for age, sex, body mass index, estimated glomerular filtration rate (eGFR), diabetes mellitus, hypertension, coronary artery disease, and smoking status. Genetic depression risk scores are presented as standardised continuous variables (Z-scores), with higher values indicating greater genetic liability to depression. P-values for overall associations (RCS Poverall) and non-linearity (RCS Pnonlinear) were derived from restricted cubic spline models and are displayed within each panel. These p-values reflect the spline analyses and are distinct from the primary multivariable-adjusted regression analyses with false discovery rate (FDR) correction presented in Supplementary Table S3

**Fig. 3:**
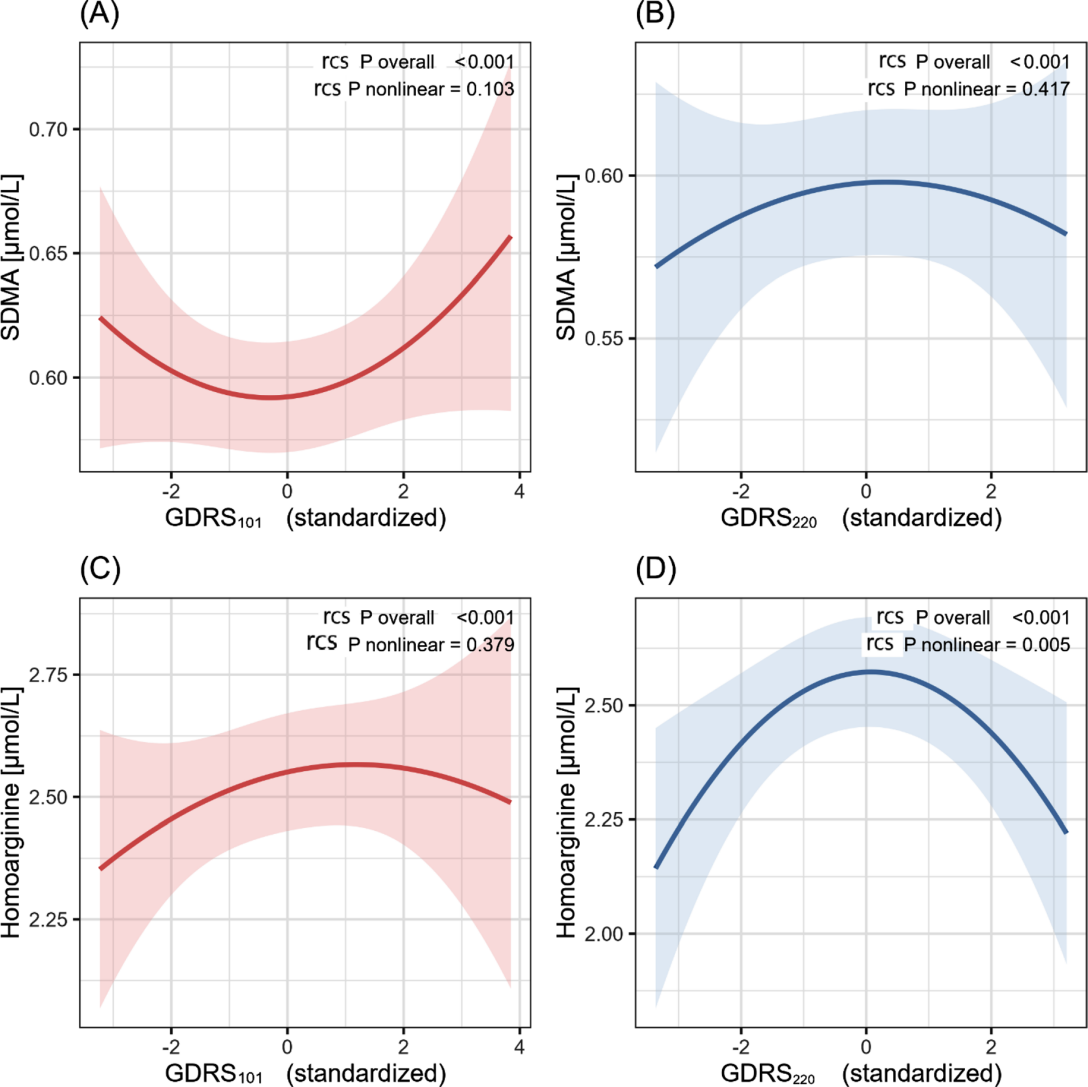
Associations between vascular-related amino acid metabolites and genetic depression risk scores assessed using restricted cubic spline (RCS) regression models. Panels (**A**) and (**C**) depict associations with GDRS_101_, whereas panels (**B**) and (**D**) depict associations with GDRS_220_. Solid lines represent model-based predicted values from multivariable-adjusted RCS models, and shaded areas indicate 95% confidence intervals. Models were adjusted for age, sex, body mass index, estimated glomerular filtration rate (eGFR), diabetes mellitus, hypertension, coronary artery disease, and smoking status. Genetic depression risk scores are presented as standardized continuous variables (Z-scores), with higher values indicating greater genetic liability to depression. P-values for overall associations (RCS P overall) and non-linearity (RCS P nonlinear) were derived from restricted cubic spline models and are displayed within each panel. These p-values reflect the spline analyses and are distinct from the primary multivariable-adjusted regression analyses with false discovery rate (FDR) correction presented in Supplementary Table S3

Restricted cubic spline analyses of selected amino acids (Fig. 2) and vascular-related metabolites (Fig. 3). For α-aminobutyric acid, a U-shaped relationship was observed for GDRS₁₀₁ (p for non-linearity = 0.091), whereas the association with GDRS₂₂₀ appeared largely linear (p for non-linearity = 0.935; Fig. 2A–B).

For α-aminoadipic acid, associations were predominantly linear, with no evidence of non-linearity (p for non-linearity = 0.942 and 0.196 for GDRS₁₀₁ and GDRS₂₂₀, respectively; Fig. 2C–D). Similarly, 1-methylhistidine showed no evidence of non-linear associations (p for non-linearity = 0.918 and 0.488; Fig. 2E–F).

In contrast, SDMA and homoarginine showed suggestive curvilinear patterns (Fig. 3). For SDMA, a modest U-shaped association was observed for GDRS₁₀₁ (p for non-linearity = 0.103), while no strong evidence of non-linearity was observed for GDRS₂₂₀ (p for non-linearity = 0.417; Fig. 3A–B). For homoarginine, a non-linear association was particularly evident for GDRS₂₂₀ (p for non-linearity = 0.005), whereas no strong evidence for non-linearity was observed for GDRS₁₀₁ (p for non-linearity = 0.379; Fig. 3C–D).

Overall, these findings suggest that while most associations were approximately linear, selected amino acids—particularly homoarginine—may exhibit non-linear patterns across the range of genetic depression risk.

### Sensitivity analyses

To account for overlap between GDRS₁₀₁ and GDRS₂₂₀, a principal component analysis was performed. The first principal component (GDRS_combined) explained 79% of the total variance (Supplementary Figure S2).

Analyses using GDRS_combined yielded consistent results, confirming the inverse association with α-aminoadipic acid. Associations involving SDMA and homoarginine showed similar trends but were attenuated after adjustment. Restricted cubic spline analyses using GDRS_combined confirmed these findings and further supported non-linear associations for selected amino acids, particularly homoarginine (Supplementary Figure S3).

## Discussion

In this study, we investigated the associations between genetic liability to depression, assessed by two genetic depression risk scores, and circulating amino acid concentrations in a well-characterised cardiovascular cohort. Using multivariable-adjusted analyses, we observed that higher genetic risk of depression was associated with lower concentrations of α-aminoadipic acid, whereas inverse associations with α-aminobutyric acid did not remain statistically significant after false discovery rate correction. Associations involving SDMA and homoarginine were attenuated after adjustment for renal function. In addition, restricted cubic spline analyses suggested predominantly linear relationships, with some evidence of non-linearity, particularly for homoarginine. Importantly, after correction for multiple testing, only the association between α-aminoadipic acid and GDRS₁₀₁ remained statistically significant, while all other associations should be interpreted as exploratory.

By modelling genetic risk as a continuous variable, our analyses enabled a nuanced assessment of dose–response relationships. These findings extend previous work on amino acid alterations in depression by focusing on genetic liability rather than clinically manifest disease. In contrast to case–control studies, this approach captures inherited susceptibility independent of disease state, treatment, or symptom severity, suggesting that the observed metabolic patterns may reflect underlying biological predisposition rather than secondary effects. Importantly, in the absence of clinical data on depression status, severity, or treatment, these findings cannot be directly compared to case–control studies of manifest depression and should be interpreted as reflecting genetic predisposition rather than clinically expressed disease.

Descriptive comparisons across GDRS strata suggested differences in amino acid profiles between individuals with lower and higher genetic liability; however, these patterns were not consistently confirmed after multivariable adjustment and correction for multiple testing. This discrepancy highlights the importance of accounting for confounding and suggests that some apparent group differences may reflect non-linear relationships or residual confounding rather than robust independent associations.

Among the investigated metabolites, α-aminobutyric acid has previously been reported to be reduced in individuals with depressive symptoms [32]. This amino acid is involved in glutathione metabolism and oxidative stress regulation [33], suggesting a potential link between depression-related genetic susceptibility and redox homeostasis. In contrast, α-aminoadipic acid has been primarily implicated in metabolic and cardiometabolic processes, and its role in depression remains less well defined. The consistent inverse association observed in the present study may therefore point towards shared metabolic pathways linking genetic susceptibility to depression with systemic metabolic regulation.

Previous studies examining amino acid profiles in depression have reported heterogeneous findings, including alterations in glutamate-, tryptophan-, methionine-, and GABA-related pathways [4, 5, 7–9, 11, 12, 14, 34]. An overview of previous studies investigating amino acid profiles in depression is provided in Table 5.

**Table 5:** Overview of studies investigating blood concentrations of amino acids in depression

Study/Type	Patients	Ethnicity	Method	Arg, His, Lys	Asp, Glu, α-AAA	Ser, Thr, Asn, Gln	Cys, Sec, Gly, Pro	Ala, Val, Ile, Leu, Nor	Met, Phe, Tyr, Trp, Kyn	GABA, Tau, Hcy, Sar, Orn, Cit	ß-Ala, BABA, Eth, Cya, α-ABA
[6] Möller OS	*n* = 26 MD *n* = 55 C	Caucasian, Danish	Plasma, ion-exchange chromatography						Trp/ LNAA↓ Tyr/ LNAA=		
[7] Mauri OS	*n* = 29 MD *n* = 28 C	Caucasian, Italian	Plasma, HPLC	Lys↑ His= Arg=	Glu↑ Asp=	Ser, Thr, Gln all=	Gly=	Ala, Val, Ile, Leu all=	Trp= Trp/LNAA↓, Phe, Tyr, Met all=	Tau↑	
[34] Almeida OS + MA	*n* = 3752	Caucasian, Australia	Plasma, fluorescence polarization immunassay							Hcy↑ TT carrier↑	
[8] Xu, OS	*n* = 25 MD *n* = 25 C	Asian, Chinese	Plasma, mass spectrometry	Lys↓					Trp↓	GABA↓	
[10] Woo OS	*n* = 68 MD *n* = 22 C	Asian, Korea	Plasma, LC-MS/MS		Glu↑			Ala↑	Met↑	GABA↑, Hcy↓ Sar↓	ß-Ala↓ BABA↓ Eth↑ Cya↓
[5] Umehara OS	*n* = 33 MD *n* = 33 C	Asian, Japan	Plasma, CE-TOFMS		Glu↑ Asp↑	Gln↓ Asn↓ Thr↑	Cys↓		Met↓ Kyn↓		Eth↓
[12] Inoshita MA	*n* = 529 MD *n* = 590 C	Caucasian, Asian	Variable		Glu↑						
[9] Ogawa, OS	*n* = 229 MD *n* = 282 C	Asian, Japan	Plasma, HPLC (set A) or LC/MS (set B)	Arg↓, Lys↓	Glu↑ Asp=	Asn↓, Thr=, Ser↓ Gln=	Gly=,	Val = Leu↓Ile = Ala=	Tyr↓, Trp↓, Phe↓, Met↓,	Tau↑	α-ABA↓, Eth=, BABA=
[11] Islam, OS	*n* = 247 MD *n* = 248 C	Asian, Bangladesh	Serum, HPLC	Arg=, His=, Lys=	Asp=, Glu=	Ser=, Thr=	Cys=, Gly=, Pro=	Ala=,Val=, Ile=, Leu=	Phe↓, Trp↓,Tyr↓, Met↓		
[15] Fan, MA	*n* = 745 MD *n* = 823 C	Caucasian, Asian	Variable	Arg=, serum Arg↓						Orn=, Cit=	
[14] Almulla, MA	*n* = 8024 MD+ bipolar *n* = 7446 C	Caucasian, Asian	Variable						Trp↓, Kyn↓		
[13] Liu, MA	n= ~ 280 post-partum MD/ C	Caucasian, Asian	Variable						Total (not free) Trp↓		
[4] Ho, OS	*n* = 70 MD *n* = 70 C	Asian, Singapore	Serum, LC-MS	Arg=, His=	Asp↑, Glu↑	Ser=	Gly↑, Pro=	Ala=, Val=, Ile=, Leu=	Met=, Phe=, Tyr=, Trp=, Kyn=	Orn=, Cit=	
[35] Uchida, OS	*n* = 13 MD *n* = 13 C	Asian, Japan	Plasma, LC-MS/MS						Trp↓, Kyn↓	GABA↓	
present Krämer, OS	*n* = 2143 genetic liability to depression	Caucasian, Germany	Serum, ion-exchange chromatography, post-column continuous reaction with ninhydrin	His (↓)	Asp (↑), Glu (↓), α-AAA↓		Cys (↑),	Val (↓), Leu (↓), Nor↑	Met (↓), Phe (↑), Tyr (↑), Trp (↓),		α-ABA↓

Original studies (OS) and meta-analyses (MA) are summarised with respect to study populations, analytical methods, and reported directions of associations. Abbreviations: Arg, arginine; His, histidine; Lys, lysine; Asp, aspartic acid; Glu, glutamic acid; α-AAA, α-aminoadipic acid; Ser, serine; Thr, threonine; Asn, asparagine; Gln, glutamine; Cys, cystine; Sec, selenocysteine; Gly, glycine; Pro, proline; Ala, alanine; Val, valine; Ile, isoleucine; Leu, leucine; Nor, norleucine; Met, methionine; Phe, phenylalanine; Tyr, tyrosine; Trp, tryptophan; Kyn, kynurenine; GABA, γ-aminobutyric acid; Tau, taurine; Hcy, homocysteine; Sar, sarcosine; Orn, ornithine; Cit, citrulline; β-ALA, beta-alanine; BABA, beta-aminobutyric acid; α-ABA, α-aminobutyric acid; Eth, ethanolamine; Cya, cystathionine; LNAA, large neutral amino acids; MD, major depression. For the present study, reported directions of association are based on multivariable-adjusted analyses; only associations remaining significant after FDR correction are considered robust

However, results have been inconsistent across studies. For instance, a South Korean study reported higher plasma concentrations of alanine, γ-aminobutyric acid, ethanolamine, and methionine, but lower concentrations of β-alanine, β-aminoisobutyric acid, cystathionine, homocysteine, O-phospho-L-serine, and sarcosine in individuals with depression [10]. Differences between studies may be explained by variation in analytical methods, disease stage (active versus remitted depression), and population characteristics such as ethnicity.

Treatment-related changes further complicate interpretation. Alterations in glutamine, kynurenine, and glutamate-related pathways have been observed following antidepressant therapy,⁠ [5] and several amino acids have been linked to disease severity [4, 17, 35]. Importantly, in the present analysis based on genetic depression risk scores, disease activity and treatment effects are not captured. Thus, the findings are more likely to reflect genetically determined metabolic susceptibility patterns rather than state-dependent alterations associated with manifest depression.

Although amino acids are frequently discussed as potential contributors to the pathophysiology of depression, it remains unclear whether observed alterations represent causal mechanisms or secondary effects. This distinction is particularly relevant in the context of genetically defined risk, where metabolic differences may reflect underlying biological predisposition rather than active disease processes.

In this cardiovascular cohort, several of the investigated amino acids have also been linked to cardiovascular disease and mortality. For example, ADMA and SDMA have been associated with adverse cardiovascular outcomes [36, 37], including in the LURIC study [38, 39]. In addition, low homoarginine concentrations and reduced arginine availability have been linked to increased cardiovascular risk and mortality [25, 40–42]. Population-based metabolomics studies have further identified phenylalanine and related metabolites as predictors of cardiovascular risk [43]. 

Additional evidence from longitudinal cohorts supports a role of amino acid metabolism in cardiometabolic outcomes. In a cohort of Japanese patients with type 2 diabetes, elevated concentrations of 3-methyl-histidine, citrulline, β-aminoisobutyric acid, and cystine, as well as lower tryptophan levels, were associated with future cardiovascular events [44]. The Framingham study further identified 2-α-aminoadipic acid as a predictor of metabolic disease development [45]. Consistent with these observations, recent analyses in LURIC (Moissl-Blanke et al., 2026, manuscript in submission) demonstrated that distinct amino acid profiles are associated with differential mortality risk. These observations are intended to contextualise the findings within the specific cardiovascular setting of the LURIC cohort and do not imply direct mechanistic links between genetic depression risk and cardiovascular outcomes.

These findings suggest that overlapping metabolic pathways may contribute to both cardiovascular and neuropsychiatric phenotypes. However, given the cross-sectional design of the present study, such relationships should be interpreted with caution and not as evidence of causal or mediating effects. In addition, survival bias in an older, high-risk cohort such as LURIC may influence the observed metabolic patterns and their relationship with genetic risk.

With respect to the kynurenine pathway, previous work has demonstrated links between tryptophan metabolites, depression, and cardiovascular outcomes [17]. However, because kynurenine measurements were available only for a subset of LURIC participants, this pathway could not be evaluated robustly in the present analysis and should be addressed in future studies.

Future research should aim to integrate genetic, metabolomic, and longitudinal clinical data to clarify the biological relevance of these associations. Approaches such as Mendelian randomisation and functional genomic analyses may help to identify causal pathways and underlying mechanisms linking depression-related genetic liability to systemic metabolism.

Taken together, our findings indicate that genetic liability to depression is associated with distinct serum amino acid profiles in a cardiovascular cohort. While these results are associative and hypothesis-generating, they support the hypothesis of shared biological pathways linking depression-related genetic architecture with metabolic regulation and cardiovascular risk.

## Strengths and limitations

All LURIC participants were of European ancestry and were recruited at a tertiary referral centre, which limits genetic heterogeneity but may reduce generalisability to other populations and ethnic groups. In addition, the cohort represents individuals with a high burden of cardiovascular disease, which may introduce selection and survival bias compared with population-based samples.

A key limitation of the present study is the absence of clinical data on depression status, severity, and treatment. Consequently, the analyses reflect metabolic correlates of genetic liability to depression rather than clinically manifest disease, limiting direct comparability with case–control studies. Furthermore, due to the cross-sectional design, causal relationships between genetic depression risk and amino acid concentrations cannot be established. Several amino acids are also strongly influenced by renal function, potentially confounding observed associations despite multivariable adjustment.

Strengths of the study include the detailed clinical and metabolic characterisation of participants, the standardised measurement of a broad panel of amino acids using established analytical methods, and the availability of high-quality genotyping data. In addition, the use of two independent genetic depression risk scores and complementary modelling approaches strengthens the robustness of the findings.

## Conclusion

In conclusion, this study demonstrates that genetic liability to depression is associated with distinct serum amino acid profiles in a cardiovascular cohort, with a consistent inverse association observed for α-aminoadipic acid. Associations involving SDMA and homoarginine were influenced by renal function, and evidence for non-linear relationships was limited to selected metabolites.

These findings are associative and hypothesis-generating and suggest that amino acid metabolism may represent a metabolic correlate of depression-related genetic susceptibility rather than a consequence of clinically manifest disease.

Future studies integrating genomic, metabolomic, and longitudinal clinical data, including approaches such as Mendelian randomisation, are needed to clarify the biological relevance and potential causal role of these pathways in the interplay between depression-related genetic liability and cardiometabolic disease.

## Supplementary Information

Below is the link to the electronic supplementary material.

Supplementary Material 1

## Data Availability

Due to the articles of the Ludwigshafen Risk and Cardiovascular Health (LURIC) Study GmbH, which require compliance with the German Data Protection Act and the consent of the study participants, data cannot be released to the public. The exploitation of the (LURIC) study database is governed by the articles of the LURIC Study GmbH (non-profit LLC), registered under number HRB 7668 at the commercial registry of Freiburg in Breisgau, Germany. According to the organisation’s articles, data may be made available to researchers upon request and approval. This procedure implies that data cannot be released to the public without formal agreement and ensures that the rules of good scientific practice are followed and that credit is given to those responsible for the design and organisation of the study. Interested researchers are invited to address their request or proposal to Kai Grunwald (Kai.Grunwald@weitnauer.net) or to the principal investigator of the LURIC study Winfried März (winfried.maerz@luric-online.de). Finally, the authors confirm that they accessed these data upon LURIC’s approval and that all other researchers can access them in the same manner.

## Abbreviations

α-AAA: α-Aminoadipic acid
α-ABA: α-Aminobutyric acid
Ala: Alanine
Arg: Arginine
Asn: Asparagine
Asp: Aspartic acid
ADMA: Asymmetric dimethylarginine
BMI: Body mass index
Cit: Citrulline
CysC: Cystatin C
Cys: Cystine
DBP: Diastolic blood pressure
eGFR: Estimated glomerular filtration rate
FDR: False discovery rate
GABA: Gamma-aminobutyric acid
GDRS₁₀₁: Genetic depression risk score (101 SNPs)
GDRS₂₂₀: Genetic depression risk score (220 SNPs)
GDRS_combined: Combined genetic depression risk score
GDRS: Genetic depression risk score
Glu: Glutamic acid
Gln: Glutamine
HDL-C: High-density lipoprotein Cholesterol
hArg: Homoarginine
Hcy: Homocysteine
hsCRP: High-sensitivity C-reactive protein
IEC: Ion-exchange chromatography
Ile: Isoleucine
Kyn: Kynurenine
Leu: Leucine
LDL-C: Low-density lipoprotein cholesterol
LURIC: Ludwigshafen Risk and Cardiovascular Health Study
MD: Major depression
Met: Methionine
NMR: Nuclear magnetic resonance
NLS: Non-linear least squares
Nor: Norleucine
NT-proBNP: N-terminal pro-B-type natriuretic peptide
OLS: Ordinary least squares
Phe: Phenylalanine
P-Ser: Phosphoserine
PCA: Principal component analysis
RCS: Restricted cubic spline
SBP: Systolic blood pressure
Ser: Serine
SD: Standard deviation
SDMA: Symmetric dimethylarginine
Thr: Threonine
Trp: Tryptophan
Tyr: Tyrosine
Val: Valine
Z: Z-score (standardised score)
