# Supplementary material for: Associations between serum amino acids and genetic liability to depression in the LURIC cohort
Source: BMC Psychiatry. 2026 Jul 17;26:539. doi: 10.1186/s12888-026-08400-7 (PMC13377875; doi:10.1186/s12888-026-08400-7)
Supplement: Supplementary file 1 — Supplementary Material 1 [file 12888_2026_8400_MOESM1_ESM.docx]

**Supplemental Material to**

**Associations between serum amino acids and genetic liability to depression in the LURIC cohort**

**Robert M. Krämer^1^, Graciela E. Delgado^2^, Marcus E. Kleber^2,3^,Daniel Duerschmied^2^, Winfried März^2,4,5^, Angela P. Moissl-Blanke^2*^**

^1^Department for Children and Adolescent Psychiatry, Central Institute for Mental Health Mannheim, Medical Faculty Mannheim of the University of Heidelberg, Mannheim, Germany

^2^Department of Medicine I (Cardiology, Hemostaseology, Medical Intensive Care), Medical Faculty Mannheim, University of Heidelberg, Mannheim, Germany.

^3^Synlab Medical Center of Human Genetics, SYNLAB Holding Deutschland GmbH, Mannheim, Germany.

^4^Synlab Academy, SYNLAB Holding Deutschland GmbH, Mannheim, Germany

^5^Department of Medicine III (Cardiology, Pneumology, Angiology), University of Heidelberg, Heidelberg, Germany

*** Correspondence:**

Dr. Angela Patricia Moissl-Blanke

Department of Medicine I

(Cardiology, Hemostaseology, Medical Intensive Care),

Medical Faculty Mannheim,

University of Heidelberg, Mannheim, Germany.

E-Mail: [angela.moissl@medma.uni-heidelberg.de](mailto:angela.moissl@medma.uni-heidelberg.de)

**Supplementary Methods**

***Correlation between genetic depression risk scores (GDRS₁₀₁ and GDRS₂₂₀)***

To assess the relationship between the two genetic depression risk scores, we calculated the Spearman rank correlation between the weighted genetic risk score derived from Howard et al. (2019; GDRS₁₀₁) and the score based on Levey et al. (2021; GDRS₂₂₀). Scatterplots with fitted linear regression lines and marginal histograms were generated using the ggplot2 and ggExtra packages in R version 4.3.1. A moderate positive correlation was observed (Spearman’s r = 0.57, p < 0.001), indicating that the two scores capture overlapping but non-identical aspects of depression-related genetic architecture.


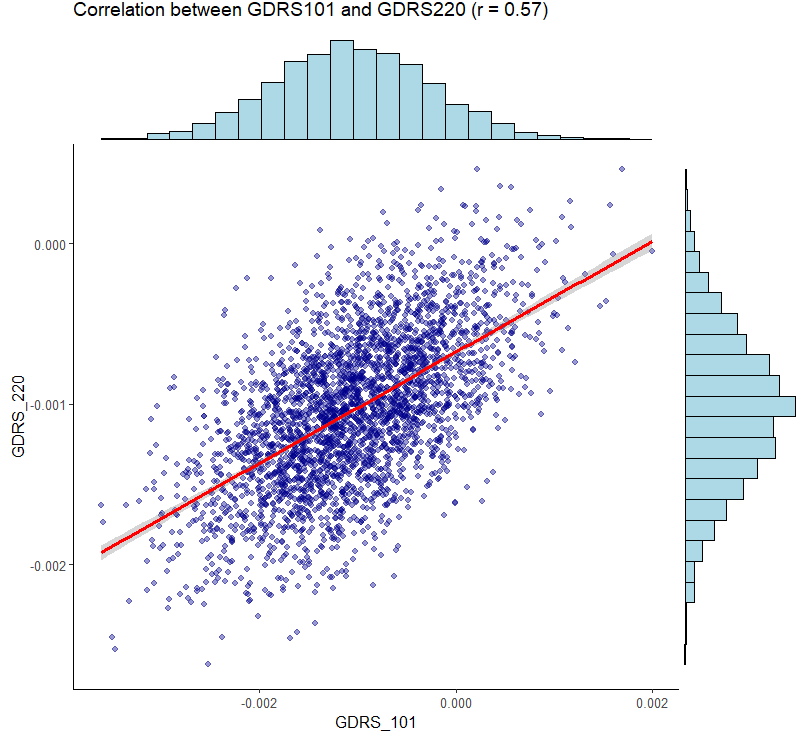


***Supplementary Figure S1:*** Correlation between GDRS₁₀₁ and GDRS₂₂₀. Scatterplot showing the association between the genetic depression risk scores derived from Howard et al. (2019; GDRS₁₀₁) and Levey et al. (2021; GDRS₂₂₀) in the LURIC cohort. Each point represents an individual participant. The red line indicates the fitted linear regression, and marginal histograms display the distributions of both scores. A moderate positive correlation was observed (Spearman’s r = 0.57, *p* < 0.001), indicating that the two scores share a common genetic component while also capturing distinct aspects of depression-related genetic architecture.

**Construction of the combined genetic depression risk score (GDRS_combined)**

To derive a combined genetic depression risk score, we performed principal component analysis (PCA) on GDRS₁₀₁ and GDRS₂₂₀. The first principal component, explaining 79% of the total variance, was retained as a summary measure of shared genetic liability to depression. This component was standardised (mean = 0, SD = 1) and used as GDRS_combined in subsequent analyses.

To account for multiple testing in the multivariable regression analyses, p-values were adjusted using the Benjamini–Hochberg false discovery rate (FDR) method across all tested amino acids (n = 10 tests).


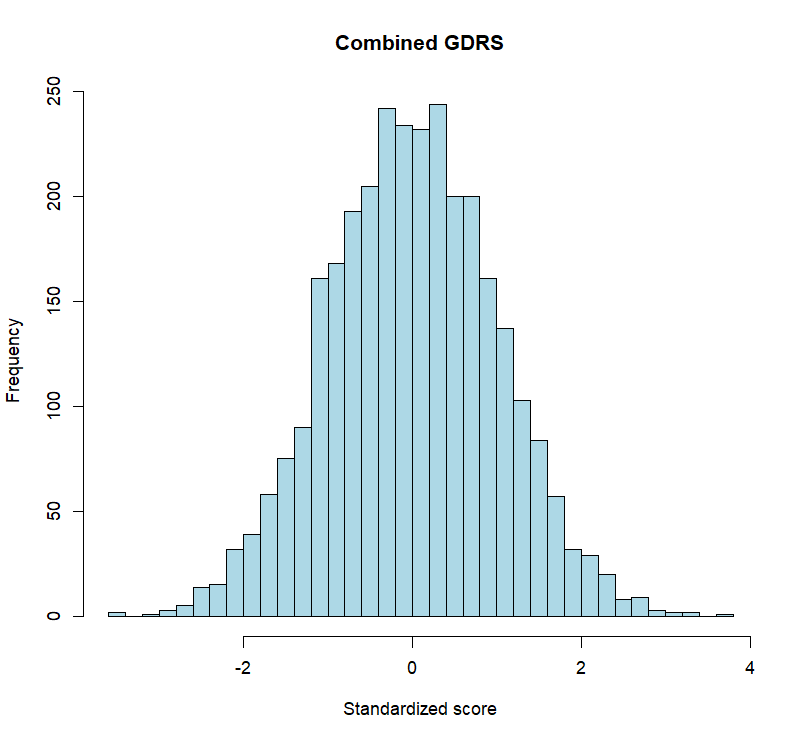


***Supplementary Figure S2.*** Distribution of the combined genetic depression risk score (GDRS_combined). Histogram showing the standardised distribution of GDRS_combined derived from a principal component analysis of GDRS₁₀₁ and GDRS₂₂₀. The first principal component explained 79% of the total variance and was standardised (mean = 0, SD = 1) to obtain GDRS_combined, which follows an approximately normal distribution.


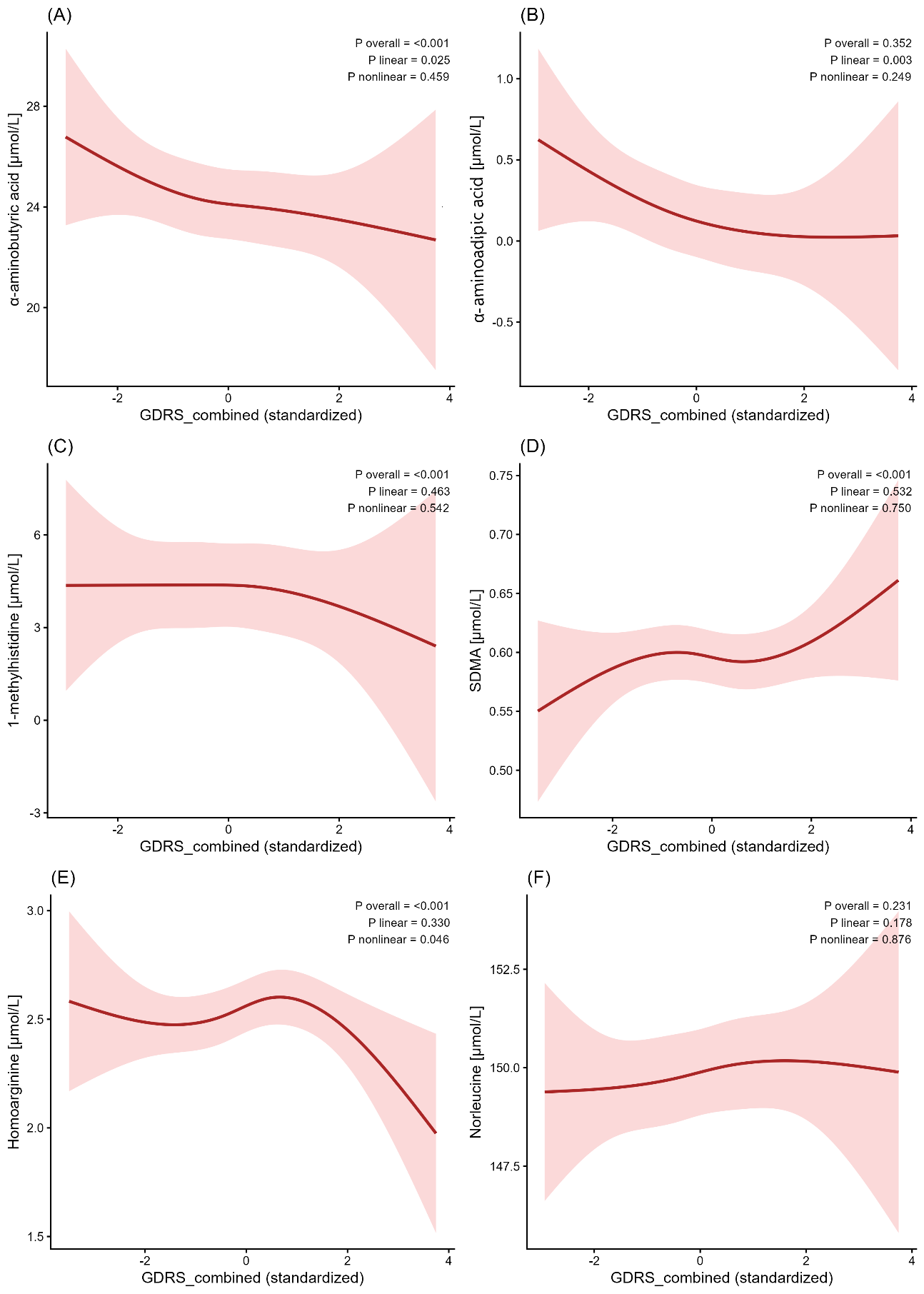


***Supplementary Figure S3.*** Associations between selected serum amino acids and the combined genetic depression risk score (GDRScombined). Solid lines represent model-based predicted values derived from multivariable-adjusted restricted cubic spline (RCS) models, and shaded areas indicate 95% confidence intervals. Models were adjusted for age, sex, body mass index, kidney function eGFR (CKD-EPI), diabetes, hypertension, coronary artery disease, and smoking status. P-values for overall, linear, and non-linear associations derived from restricted cubic spline (RCS) models are displayed within each panel. These p-values reflect the spline analyses and are distinct from the primary multivariable-adjusted regression analyses with false discovery rate (FDR) correction presented in Supplementary Table S3.

**Supplementary Tables:**

***Supplementary Table 1*:** Correlation of amino acid concentrations with the genetic depression risk score **GDRS_101** given for the total cohort and in addition for the lower and higher median split.

| Variables | GDRS101 | | | | | | |
| --- | --- | --- | --- | --- | --- | --- | --- |
|  | **GDRS total** | **σ** | **GDRS Low** | **σ** | **GDRS High** | **σ** |  |
| Aspartic acid, µmol/L | -0.001 | ns | 0.058 | 0.06 | 0.010 | ns |  |
| Threonine, µmol/L | 0.01 | ns | -0.031 | ns | 0.066 | * |  |
| Serine, µmol/L | -0.011 | ns | -0.036 | ns | 0.026 | ns |  |
| Asparagine, µmol/L | 0.014 | ns | 0.015 | ns | 0.028 | ns |  |
| Glutamic acid, µmol/L | 0.025 | ns | 0.018 | ns | 0.042 | ns |  |
| Glutamine, µmol/L | -0.003 | ns | -0.032 | ns | -0.061 | * |  |
| α-Aminobutyric acid, µmol/L | -0.069 | ** | -0.027 | ns | 0.013 | ns |  |
| Proline, µmol/L | 0.019 | ns | 0.016 | ns | 0.026 | ns |  |
| Glycine, µmol/L | -0.018 | ns | -0.022 | ns | -0.038 | ns |  |
| Alanine, µmol/L | 0.010 | ns | -0.014 | ns | 0.049 | ns |  |
| Citrulline, µmol/L | 0.006 | ns | -0.060 | 0.051 | -0.010 | ns |  |
| α-Aminoadipic acid, µmol/L | -0.05 | * | -0.063 | 0.041 | 0.034 | ns |  |
| Valine, µmol/L | -0.021 | ns | -0.058 | 0.061 | 0.038 | ns |  |
| Cystine, µmol/L | -0.032 | ns | -0.023 | ns | -0.015 | ns |  |
| Methionine, µmol/L | -0.012 | ns | -0.060 | 0.052 | 0.050 | ns |  |
| Isoleucine, µmol/L | -0.013 | ns | -0.037 | ns | 0.016 | ns |  |
| Leucine, µmol/L | -0.032 | ns | -0.075 | * | 0.024 | ns |  |
| Norleucine, µmol/L | 0.047 | * | 0.064 | * | 0.037 | ns |  |
| Tyrosine, µmol/L | 0.033 | ns | 0.016 | ns | 0.046 | ns |  |
| Phenylalanine, µmol/L | -0.127 | ns | -0.064 | * | 0.021 | ns |  |
| Histidine, µmol/L | 0.027 | ns | -0.060 | 0.053 | -0.004 | ns |  |
| 3-Methylhistidine, µmol/L | -0.017 | ns | -0.023 | ns | 0.001 | ns |  |
| 1-Methylhistidine, µmol/L | -0.014 | ns | 0.005 | ns | 0.009 | ns |  |
| Tryptophan, µmol/L | 0.040 | 0.065 | 0.033 | ns | 0.080 | ** |  |
| Ornithine, µmol/L | -0.001 | ns | -0.051 | ns | 0.001 | ns |  |
| Lysine, µmol/L | -0.008 | ns | -0.039 | ns | 0.039 | ns |  |
| Arginine, µmol/L | 0.021 | ns | -0.011 | ns | -0.006 | ns |  |
| Homoarginine, µmol/L | 0.052 | ** | 0.044 | ns | 0.044 | ns |  |

*Correlation coefficient (σ= significance****)****, ^a^P value < 0.05 was considered statistically significant; * statistically significant correlation at two-sided 0.05 level, ** statistically significant correlation at two-sided 0.01 level.*

***Supplementary Table* 2:** Correlation of amino acid concentrations with the genetic depression risk score **GDRS_220** given for the total cohort, and in addition for the lower and higher median split.

| Variables | GDRS_220 | | | | | |
| --- | --- | --- | --- | --- | --- | --- |
|  | **GDRS**  **Total** | **σ** | **GDRS Low** | **σ** | **GDRS High** | **σ** |
| Aspartic acid, µmol/L | -0.002 | ns | -0.013 | ns | -0.014 | ns |
| Threonine, µmol/L | 0.031 | ns | 0.014 | ns | 0.013 | ns |
| Serine, µmol/L | 0.003 | ns | -0.015 | ns | 0.004 | ns |
| Asparagine, µmol/L | 0.003 | ns | -0.004 | ns | 0.007 | ns |
| Glutamic acid, µmol/L | 0.018 | ns | 0.046 | ns | 0.005 | ns |
| Glutamine, µmol/L | 0.006 | ns | 0.014 | ns | 0.012 | ns |
| α-Aminobutyric-acid, µmol/L | -0.043 | * | -0.016 | ns | -0.027 | ns |
| Proline, µmol/L | 0.014 | ns | 0.039 | ns | 0.006 | ns |
| Glycine, µmol/L | -0.021 | ns | -0.013 | ns | -0.009 | ns |
| Alanine, µmol/L | 0.036 | ns | 0.043 | ns | 0.024 | ns |
| Citrulline, µmol/L | -0.005 | ns | -0.004 | ns | 0.032 | ns |
| α-Aminoadipic acid, µmol/L | -0.043 | * | -0.026 | ns | -0.060 | * |
| Valine, µmol/L | -0.009 | ns | 0.001 | ns | -0.004 | ns |
| Cystine, µmol/L | -0.033 | ns | -0.078 | ns | 0.012 | ns |
| Methionine, µmol/L | 0.013 | ns | 0.017 | ns | -0.027 | ns |
| Isoleucine, µmol/L | 0.013 | ns | 0.031 | ns | -0.016 | ns |
| Leucine, µmol/L | -0.004 | ns | 0.001 | ns | -0.021 | ns |
| Norleucine, µmol/L | 0.003 | ns | 0.042 | ns | 0.021 | ns |
| Tyrosine, µmol/L | 0.018 | ns | 0.016 | ns | 0.003 | ns |
| Phenylalanine, µmol/L | -0.022 | ns | -0.027 | ns | -0.038 | ns |
| Histidine, µmol/L | -0.004 | ns | 0.003 | ns | 0.031 | ns |
| 3-Methylhistidine, µmol/L | -0.024 | ns | -0.028 | ns | -0.031 | ns |
| 1-Methylhistidine, µmol/L | -0.027 | ns | 0.004 | ns | -0.003 | ns |
| Tryptophan, µmol/L | 0.014 | ns | 0.038 | ns | 0.005 | ns |
| Ornithine, µmol/L | -0.008 | ns | -0.012 | ns | 0.007 | ns |
| Lysine, µmol/L | -0.016 | ns | 0.018 | ns | -0.031 | ns |
| Arginine, µmol/L | 0.034 | ns | 0.016 | ns | -0.014 | ns |
| Homoarginine, µmol/L | 0.035 | 0.051 | -0.052 | * | -0.053 | * |

*Correlation coefficient (σ= significance), ^a^P value < 0.05 was considered statistically significant; * statistically significant correlation at two-sided 0.05 level, ** statistically significant correlation at two-sided 0.01 level.*

**Supplementary Table 3:** Multivariable-adjusted associations between genetic depression risk scores and selected serum amino acids, including FDR-adjusted p-values

| **Amino acid** | **GDRS₁₀₁ β (95% CI)** | **p-value** | **FDR** | **GDRS₂₂₀ β (95% CI)** | **p-value** | **FDR** |
| --- | --- | --- | --- | --- | --- | --- |
| α-Aminoadipic acid | −0.097 (−0.161 to −0.033) | 0.003 | 0.030 | −0.076 (−0.141 to −0.011) | 0.021 | 0.105 |
| α-Aminobutyric acid | −0.404 (−0.803 to −0.005) | 0.047 | 0.118 | −0.409 (−0.813 to −0.006) | 0.047 | 0.118 |
| 1-Methylhistidine | −0.093 (−0.481 to 0.294) | 0.637 | 0.758 | −0.166 (−0.558 to 0.225) | 0.405 | 0.649 |
| SDMA | 0.002 (−0.004 to 0.009) | 0.454 | 0.649 | 0.001 (−0.005 to 0.008) | 0.717 | 0.758 |
| Homoarginine | 0.025 (−0.010 to 0.060) | 0.154 | 0.308 | 0.006 (−0.030 to 0.041) | 0.758 | 0.758 |

*P-values were adjusted for multiple testing using the Benjamini–Hochberg false discovery rate (FDR) method across all amino acids*

*(n = 10 tests). Statistically significant results after FDR correction (FDR < 0.05) are highlighted in bold.*
